# Biochemical alterations in endocrine and inflammatory pathways following breast cancer surgery: Laboratory evidence and clinical implications

**DOI:** 10.5937/jomb0-63067

**Published:** 2026-04-15

**Authors:** Yafang Luo, Jinglan Xu, Hui Chen

**Affiliations:** 1 Hangzhou Hospital of Traditional Chinese Medicine, Department of Breast Diseases, Hangzhou, China

**Keywords:** endocrine biomarkers, inflammatory mediators, IL-6, CRP, TNF-α, estradiol, breast cancer, postoperative nursing care, biochemical profiling, endokrini biomarkeri, inflamatorni medijatori, IL-6, CRP, TNF-α, estradiol, rak dojke, postoperativna nega, biohemijsko profilisanje

## Abstract

**Background:**

Breast cancer surgery induces substantial physiological stress that disrupts endocrine regulation and triggers systemic inflammation. However, the biochemical dynamics underlying these perioperative alterations remain insufficiently characterized. This study investigated laboratory-based endocrine and inflammatory biomarker changes following breast cancer surgery and explored their clinical significance.

**Methods:**

A total of 120 breast cancer patients were evaluated. Serum estradiol (E2), progesterone (P), interleukin-6 (IL-6), C-reactive protein (CRP), and tumor necrosis factor-a (TNF-<span style="color: rgb(32, 33, 34); font-family: sans-serif; font-size: 16px; background-color: rgb(248, 249, 250);">α</span>) were measured preoperatively and on postoperative day 7 using validated ELISA assays with strict analytical quality control. Clinical outcomes - including psychological status, cognitive recovery, quality of life, and postoperative complications - were assessed as secondary indicators to explore potential biochemical-clinical associations.

**Results:**

Postoperative endocrine suppression was evident, with significant reductions in E2 and P levels (P &lt; 0.05), reflecting acute stress-related modulation of hypothalamic-pituitary-gonadal (HPG) axis activity. IL-6, CRP and TNF-<span style="color: rgb(32, 33, 34); font-family: sans-serif; font-size: 16px; background-color: rgb(248, 249, 250);">α</span> declined significantly postoperatively (all P &lt; 0.001), demonstrating resolution of acute-phase inflammatory activation. Greater biochemical stabilization was associated with improved emotional scores, cognitive function, and quality-of-life indices, as well as a reduced incidence of postoperative complications.

**Conclusions:**

Breast cancer surgery induces characteristic biochemical changes involving endocrine suppression and systemic inflammatory activation. Monitoring hormonal and inflammatory biomarkers provides valuable insight into postoperative physiological stress, recovery mechanisms, and potential risk stratification. These findings support integrating laboratory-based biochemical assessments into postoperative management frameworks for breast cancer patients.

## Abstract (sr)

**Uvod:**

Operacija raka dojke izaziva značajan fiziološki stres koji remeti endokrinu regulaciju i pokreće sistemsku upalu. Međutim, biohemijska dinamika koja leži u osnovi ovih perioperativnih promena ostaje nedovoljno okarakterisana. Ova studija je istraživala laboratorijski utvrđene promene endokrinih i inflamatornih biomarkera nakon operacije raka dojke i istraživala njihov klinički značaj.

**Metode:**

Ukupno je procenjeno 120 pacijentkinja obolelih od raka dojke. Serumski estradiol (E2), progesteron (P), interleukin-6 (IL-6), C-reaktivni protein (CRP) i faktor tumorske nekroze-a (TNF-<span style="color: rgb(32, 33, 34); font-family: sans-serif; font-size: 16px; background-color: rgb(248, 249, 250);">α</span>) mereni su preoperativno i 7. dana nakon operacije korišćenjem validiranih ELISA testova uz strogu analitičku kontrolu kvaliteta. Klinički ishodi - uključujući psihološki status, kognitivni oporavak, kvalitet života i postoperativne komplikacije - procenjeni su kao sekundarni indikatori za istraživanje potencijalnih biohemijsko-kliničkih veza.

**Rezultati:**

Postoperativna endokrina supresija bila je evidentna, sa značajnim smanjenjem nivoa E2 i P (P &lt; 0,05), što odražava modulaciju aktivnosti hipotalamusno-hipofizno-gonadne (HPG) ose povezanu sa akutnim stresom. IL-6, CRP i TNF-<span style="color: rgb(32, 33, 34); font-family: sans-serif; font-size: 16px; background-color: rgb(248, 249, 250);">α</span> su značajno opali postoperativno (svi P &lt; 0,001), što pokazuje rešavanje inflamatorne aktivacije akutne faze. Veća biohemijska stabilizacija bila je povezana sa poboljšanim emocionalnim rezultatima, kognitivnim funkcijama i indeksima kvaliteta života, kao i smanjenom učestalošću postoperativnih komplikacija.

**Zaključak:**

Hirurško lečenje raka dojke izaziva karakteristične biohemijske promene koje uključuju endokrinu supresiju i sistemsku inflamatornu aktivaciju. Praćenje hormonskih i inflamatornih biomarkera pruža dragocen uvid u postoperativni fiziološki stres, mehanizme oporavka i potencijalnu stratifikaciju rizika. Ovi nalazi podržavaju integraciju laboratorijskih biohemijskih procena u postoperativne okvire lečenja pacijenata sa rakom dojke.

## Introduction

Breast cancer ranks among the most prevalent malignancies in women globally, posing a substantial burden on public health [1]. While surgical resection serves as the cornerstone of treatment for most patients, it elicits a cascade of systemic biochemical responses that transcend the immediate surgical site. Mounting evidence indicates that perioperative stress profoundly disrupts endocrine homeostasis, immune regulation, and inflammatory pathways—processes integral to tumor progression, wound healing, and overall recovery [2].

Surgical trauma stimulates the hypothalamic-pituitary-adrenal (HPA) axis, thereby modulating the hypothalamic-pituitary-gonadal (HPG) axis through stress-mediated neuroendocrine interactions. This interplay manifests as fluctuations in circulating estradiol (E2) and progesterone (P), hormones with multifaceted roles beyond reproduction [2]. In the context of breast cancer, E2 and P influence the tumor microenvironment, immune modulation, cytokine production, and metabolic processes, rendering postoperative hormonal shifts a vital biochemical marker of physiological stress and convalescence [3]. Disruptions in these endocrine pathways may contribute to postoperative fatigue, cognitive impairment, emotional instability, and altered immune function.

In parallel, surgical manipulation induces a robust acute-phase inflammatory response characterized by elevated concentrations of interleukin-6 (IL-6), tumor necrosis factor-α (TNF-α), and C-reactive protein (CRP). IL-6 acts as a central mediator that stimulates hepatic CRP synthesis through the IL-6/STAT3 signaling axis, whereas TNF-α represents an upstream regulator of pro-inflammatory gene transcription and NF-kB activation [3]. Persistent postoperative inflammation is associated with increased metabolic stress, delayed wound healing, oxidative imbalance, and adverse functional outcomes. Therefore, dynamic assessment of inflammatory markers provides essential diagnostic and prognostic value in laboratory medicine.

Despite the acknowledged biochemical importance of these endocrine and inflammatory mediators, few studies have systematically examined the integrated biochemical shifts in breast cancer patients following surgery. The current literature largely focuses on clinical recovery approaches [4], leaving the underlying biochemical pathways that regulate postoperative physiological equilibrium underexplored. A deeper understanding of surgery's impact on hormone levels, cytokine profiles, and acute-phase reactants is crucial for unraveling recovery mechanisms and enhancing the accuracy of postoperative monitoring and risk stratification [5].

This study therefore investigated perioperative biochemical changes in key endocrine and inflammatory markers—such as E2, P IL-6, CRP and TNF-α - in breast cancer patients undergoing surgery. By delineating these laboratory-derived alterations and their correlations with clinical outcomes, we aimed to offer mechanistic insights into postoperative stress responses and advocate for the incorporation of biochemical monitoring into routine postoperative care.

## Materials and methods

### General information

A total of 120 female patients who underwent surgical treatment for breast cancer at our institution between June 2023 and April 2025 were consecutively recruited. Patients were randomly assigned to an experimental group or a control group using a computer-generated random number table, with 60 cases in each group. Baseline demographic and clinical characteristics - including age, disease duration, and body mass index (BMI) - showed no statistically significant differences between the two groups (P > 0.05), ensuring comparability at enrollment. Detailed baseline parameters are summarized in Table 1.

**Table 1:** Comparison of baseline characteristics between the two groups (x̄ ± s, n(%)).

Group	n	Age (years)	Disease duration (years)	BMI (kg/m^2^)
Experimental group	60	54.91±4.77	4.83±1.19	20.40±2.79
Control group	60	55.04±5.06	4.77±1.22	20.52±2.57
t		0.145	0.273	0.245
P		0.885	0.785	0.807

### Eligibility criteria

Participants were recruited if they met the established diagnostic criteria for breast cancer [6], with all cases verified through histopathological examination. Enrollment was limited to individuals with intact cognitive function and the capacity to comply with perioperative protocols, and written informed consent was obtained from both patients and their families prior to involvement. Exclusion criteria encompassed severe cardiovascular or cerebrovascular conditions, inadequately controlled diabetes, documented distant metastases to the liver, lungs, or bones, major psychiatric disorders that could compromise adherence, or hematologic/immune disorders potentially confounding biomarker reliability or postoperative recovery. These criteria were designed to minimize confounding influences from comorbidities on endocrine or inflammatory pathways, thereby ensuring the integrity of biochemical assessments.

### Study design

All enrolled patients underwent standardized perioperative care. The control group adhered to conventional postoperative protocols, encompassing vital-sign monitoring, wound management, and general recovery instructions. To assess the impact of enhanced recovery strategies on biochemical pathways, the experimental group received a supplementary comprehensive management regimen alongside routine care. This regimen included targeted psychological support to mitigate perioperative stress, personalized nutritional counseling aligned with metabolic and gastrointestinal needs, and graduated rehabilitation exercises to improve circulation, mobility, and physiological stability post-surgery. These interventions were administered continuously for six months to guarantee sufficient exposure and uniformity. While these clinical components were employed to evaluate secondary outcomes, the study's primary emphasis remained on laboratory-derived biochemical assessments.

### Biochemical measurements

Biochemical indicators represented the primary outcomes of the study. Venous blood samples were collected at two standardized time points - 24 hours before surgery and on postoperative day 7 - to minimize the influence of circadian rhythm and dietary variation on biomarker fluctuations. Fasting morning samples were obtained from all participants, allowed to clot at room temperature, and subsequently centrifuged at 3,000 rpm for 10 minutes to isolate serum. The resulting serum aliquots were immediately transferred into sterile cryovials and stored at -80°C to prevent degradation and preserve molecular stability prior to biochemical analysis.

Serum estradiol (E2) and progesterone (P), key endocrine markers implicated in breast cancer biology and stress physiology, were quantified using validated enzyme-linked immunosorbent assay (ELISA) kits. Additionally, concentrations of interleukin-6 (IL-6), C-reactive protein (CRP), and tumor necrosis factor-α (TNF-α) were measured as representative inflammatory biomarkers associated with acute-phase responses and systemic immune activation. All assays strictly adhered to the manufacturers' protocols. To ensure analytical precision and alignment with laboratory medicine standards, each sample was analyzed in duplicate, and standard calibration curves were generated for every batch. Internal quality controls were incorporated to assess assay reliability, and batch-to-batch consistency checks were performed to minimize analytical variation. These quality-assurance procedures ensured the accuracy, reproducibility, and interpretability of biochemical data in accordance with the expectations of medical biochemistry research.

### Clinical and functional assessments

While the study's primary focus was on biochemical responses, supplementary clinical metrics were gathered to investigate possible links between laboratory alterations and functional recovery. Emotional well-being was quantified using the Self-Rating Anxiety Scale (SAS) and Self-Rating Depression Scale (SDS), which offer validated indices of psychological distress. Cognitive function was appraised via the Mini-Mental State Examination (MMSE) and Montreal Cognitive Assessment (MoCA), established instruments for neurocognitive evaluation. Health-related quality of life was gauged with the SF-36 questionnaire, encompassing domains such as physical function, mental health, general vitality, and social integration. Postoperative adverse events - encompassing nausea/vomiting, infections, malnutrition, and hypoglycemia - were meticulously tracked throughout the follow-up to assess clinical safety and explore biochemical-clinical interconnections.

### Statistical analysis

All statistical analyses were conducted using SPSS version 26.0. Continuous variables were expressed as mean±standard deviation (±s). Between-group comparisons of continuous variables were performed using the independent-samples t-test, whereas categorical variables were presented as frequencies and percentages and analyzed using the chi-square test (χ^2^). A two-tailed P value < 0.05 was defined as statistically significant. The chosen statistical approach ensured rigorous and appropriate evaluation of both biochemical and clinical data.

## Results

### Comparison of hormone levels between the two groups before and after intervention

Baseline serum estradiol (E2) and progesterone (P) concentrations did not differ significantly between the two groups (P > 0.05), confirming comparability prior to treatment. Following postoperative intervention, both biomarkers exhibited a marked decline in all patients, consistent with expected postoperative suppression of hypothalamic-pituitary-gonadal activity. However, the extent of hormonal reduction varied substantially between groups. The experimental group demonstrated significantly lower postoperative E2 and P levels than the control group (P < 0.05), indicating a more pronounced modulation of endocrine recovery dynamics. Detailed biochemical values are provided in Table 2.

**Table 2:** Comparison of hormone levels between the experimental and control groups before and after intervention (±s). Note: Compared with pre-intervention, P < 0.05.

Group	n	E2 (pmol/L)		P (ng/L)	
		Before	After	Before	After
Experimental group	60	155.30±14.76	76.05±7.26*	54.59±5.24	24.72±2.82*
Control group	60	154.70±14.74	95.08±8.03*	55.40±5.28	36.42±3.56*
t		0.223	13.617	0.843	19.955
P		0.824	<0.001	0.401	<0.001

### Comparison of inflammatory biomarkers between the two groups before and after intervention

Serum IL-6, CRP and TNF-α levels were comparable at baseline in the two groups (P > 0.05). Postoperative measurements revealed significant reductions in all inflammatory biomarkers within both groups, reflecting the gradual resolution of acute inflammatory activation following surgery. Nonetheless, the magnitude of decline was consistently greater in the experimental group, which exhibited significantly lower postoperative IL-6, CRP and TNF-α levels compared to the control group (all P < 0.05). These findings suggest that postoperative physiological stabilization, as reflected by laboratory-based inflammatory indicators, occurred more efficiently in the experimental cohort. Corresponding biochemical data are shown in Table 3.

**Table 3:** Comparison of Inflammatory Biomarker Levels between Experimental and Control Groups before and after Intervention (±s). Note: Compared with pre-intervention, P < 0.05.

Group	n	IL-6 (ng/L)		CRP (mg/L)		TNF-α (ng/mL)	
		Before	After	Before	After	Before	After
Experimental group	60	82.75±8.84	32.20±3.47*	32.98±3.39	18.60±1.39*	25.29±3.08	8.49±1.14*
Control group	60	82.69±8.73	48.17±4.22*	32.81±3.35	25.12±2.13*	25.03±3.02	13.29±1.85*
t		0.037	22.642	0.276	19.857	0.467	17.11
P		0.971	<0.001	0.783	<0.001	0.641	<0.001

### Comparison of SAS and SDS scores between the two groups before and after intervention

Although endocrine and inflammatory biomarkers were the primary study variables, emotional status was evaluated to explore potential correlations between biochemical stabilization and psychological recovery. Initial SAS and SDS scores did not differ significantly between the two groups (P > 0.05). After the six-month intervention period, both scores showed significant reductions in all patients. The experimental group, however, demonstrated markedly lower SAS and SDS values than the control group (P < 0.05), suggesting that improved biochemical homeostasis may contribute to enhanced psychological resilience. Detailed results are presented in Table 4.

**Table 4:** Comparison of SAS and SDS Scores before and after intervention (±s, points). Note: Compared with pre-intervention, P < 0.05.

Group	n	SAS Score		SDS Score	
		Before	After	Before	After
Experimental group	60	72.95±6.95	46.25±4.79	70.11±6.65	45.14±4.84
Control group	60	72.74±6.83	60.05±5.32	70.43±6.42	58.51±5.61
t		0.167	14.932	0.268	13.978
P		0.868	<0.001	0.789	<0.001

### Comparison of cognitive function between the two groups before and after intervention

Baseline cognitive function, evaluated via the MMSE and MoCA scales, showed no significant differences between the groups (P > 0.05). Post-intervention, both cohorts demonstrated notable improvements in cognitive performance. However, the experimental group attained superior postoperative MMSE and MoCA scores compared to the control group (P < 0.05). These results suggest that neurocognitive recovery could be modulated by postoperative biochemical stabilization and the attenuation of systemic stress responses. Detailed data are presented in Table 5.

**Table 5:** Comparison of cognitive function scores between experimental and control groups (±s, points).

Group	n	MMSE Score		MoCA Score	
		Before	After	Before	After
Experimental group	60	20.16±2.17	28.64±2.76*	20.72±2.12	26.77±2.70*
Control group	60	20.77±2.14	25.06±2.39*	20.54±2.16	23.56±2.43*
t		1.55	7.595	0.461	6.845
P		0.124	<0.001	0.646	<0.001

### Comparison of quality-of-life scores between the two groups before and after intervention

Before intervention, physical function, psychological function, general health, and social function scores were similar between the two groups (P > 0.05). At the six-month evaluation, all four domains demonstrated significant improvement in both cohorts. Importantly, the experimental group achieved consistently higher scores across all quality-of-life dimensions compared with the control group (P < 0.05). These improvements corresponded with more favorable endocrine and inflammatory biomarker profiles in the experimental group. Detailed outcomes are provided in Table 6.

**Table 6:** Comparison of Quality-of-Life Scores between Experimental and Control Groups before and after Intervention (±s, points).

Group	n	Physical Function		Psychological Function		General Health		Social Function	
		Before	After	Before	After	Before	After	Before	After
Experimental group	60	50.21±<br>5.32	72.86±<br>6.66*	50.80±<br>5.07	73.02±<br>6.73*	51.20±<br>5.28	70.78±<br>6.82*	52.56±<br>4.79	70.08±<br>6.64*
Control group	60	50.49±<br>5.30	63.23±<br>5.90*	50.06±<br>5.10	62.77±<br>5.46*	51.11±<br>5.12	64.40±<br>5.99*	52.72±<br>4.77	64.66±<br>5.72*
t		0.289	8.384	0.797	9.161	0.095	5.444	0.183	4.79
P		0.773	<0.001	0.427	<0.001	0.924	<0.001	0.855	<0.001

### Comparison of postoperative adverse reactions between the two groups

The incidence of postoperative adverse reactions was significantly lower in the experimental group (5.00%) than in the control group (18.33%), with x^2^ = 3.962 and P = 0.047. Reported complications included nausea or vomiting, infection, malnutrition, and hypoglycemia. The reduced complication rate in the experimental group aligns with its more pronounced stabilization of biochemical indicators, particularly inflammatory markers, suggesting a link between laboratory biomarker improvement and reduced postoperative risk. Specific adverse-event distributions are listed in Table 7.

**Table 7:** Comparison of adverse reactions between experimental and control groups (n (%)).

Group	n	Nausea/Vomiting	Infection	Malnutrition	Hypoglycemia	Total Incidence
Experimental<br>group	60	1 (1.67)	1 (1.67)	0 (0.00)	1 (1.67)	3 (5.00)
Control group	60	3 (5.00)	3 (5.00)	2 (3.33)	3 (5.00)	11 (18.33)
χ^2^						3.962
P						0.047

## Discussion

Breast cancer arises from a complex interplay of genetic, endocrine, and environmental factors, with key susceptibility genes like BRCA1 and BRCA2 exerting substantial influence [7]. Imbalances in hormonal milieu - particularly prolonged estrogen exposure - drive mammary epithelial proliferation and act as pivotal biochemical catalysts for malignant progression. Environmental and lifestyle elements, such as diet, alcohol intake, sedentary behavior, and chronic stress, can exacerbate endocrine disruptions by perturbing hypothalamic-pituitary-adrenal (HPA) axis activity and compromising immune surveillance [8]. Together, these elements underscore the multifaceted biochemical and molecular pathways in breast cancer pathogenesis, highlighting the need to elucidate postoperative biochemical dynamics.

Surgical intervention constitutes a profound physiological stressor, triggering cascades of neuroendocrine and inflammatory responses. In the acute postoperative phase, cytokines including IL-6, CRP and TNF-α undergo rapid upregulation, signifying acute-phase reactions and systemic immune activation. These biomarkers serve as critical tools in laboratory medicine for assessing tissue damage, inflammation, and postoperative risks. Prolonged elevations may disrupt metabolic equilibrium, hinder wound healing, and adversely affect oncologic outcomes. Thus, delineating the temporal trajectory of these markers post-surgery is vital for comprehending physiological stress and recovery processes [9][10][11][12][13][14][15].

In the present study, patients who received the comprehensive postoperative management strategy exhibited significantly greater reductions in E2, P IL-6, CRP and TNF-α compared with those receiving routine care. Although such interventions are traditionally evaluated from psychosocial or nursing perspectives, the current findings provide clear evidence that these strategies also confer meaningful biochemical benefits, supporting the hypothesis that postoperative recovery is closely intertwined with endocrine and inflammatory pathways [16][17][18][19][20].

The marked declines in estradiol and progesterone levels in the experimental group indicate that stabilizing psychological well-being, nutritional status, and circadian rhythms may regulate HPA-HPG axis interactions, thereby modulating postoperative endocrine dynamics [21]. Psychological stress disrupts cortisol release and sympathetic activation, consequently impairing gonadal hormone synthesis. Enhanced nutrition could optimize hepatic hormone clearance, while targeted rehabilitation might restore autonomic equilibrium and improve vascular function, collectively fostering robust endocrine recovery.

Likewise, the substantial reductions in IL-6, CRP and TNF-α in the experimental group point to accelerated resolution of systemic inflammation. IL-6 drives the acute-phase response and induces hepatic CRP synthesis, whereas TNF-α facilitates NF-kB - mediated cytokine cascades, apoptosis control, and metabolic perturbations. Factors such as psychological strain, suboptimal nutrition, and immobility are known to amplify these cytokines; thus, multifaceted interventions addressing them may dampen inflammatory pathways and bolster postoperative biochemical homeostasis [22].

Although emotional status, cognitive function, and quality-of-life scores served as secondary outcomes, the observed improvements in the experimental group likely reflect downstream effects of biochemical stabilization. Reduced systemic inflammation has been associated with improved neurocognitive outcomes and decreased fatigue, whereas endocrine normalization contributes to psychological regulation and metabolic balance [23][24]. These findings support the concept that biochemical processes - rather than psychosocial factors alone - play a fundamental role in postoperative functional recovery.

Moreover, the markedly reduced incidence of postoperative adverse events in the experimental group corresponds to the enhancements in endocrine and inflammatory biomarkers. Heightened cytokine profiles and endocrine imbalances are established contributors to complications like infections, metabolic derangements, and delayed tissue regeneration. Thus, the biochemical advancements documented in this study may elucidate the diminished postoperative risks in the experimental cohort [25].

In summary, this investigation emphasizes the dual clinical and laboratory value of biochemical monitoring in breast cancer surgery. The results illustrate that interventions targeting neuroendocrine and inflammatory pathways yield quantifiable biochemical shifts, which in turn foster superior postoperative outcomes. This underscores the imperative to incorporate endocrine and inflammatory biomarker evaluations into postoperative care protocols, thereby refining clinical decision-making and advancing personalized recovery approaches.

## Conclusion

In conclusion, this study provides clear evidence that comprehensive postoperative management in breast cancer patients leads to significant biochemical improvements, reflected by reductions in estradiol, progesterone, IL-6, CRP, and TNF-α. These findings indicate that postoperative physiological recovery is closely linked to endocrine regulation and inflammatory resolution. Moreover, the parallel improvements in psychological status, cognitive function, quality of life, and complication rates suggest that biochemical stabilization may have broader systemic effects extending beyond laboratory parameters.

Taken together, the results support the incorporation of endocrine and inflammatory biomarker monitoring into postoperative assessment models. Future studies should further investigate the molecular pathways linking postoperative interventions with biochemical recovery, with the aim of developing precision-based, laboratory-guided postoperative management strategies for breast cancer patients.

## Dodatak

### Limitations

This study has several limitations, including its single-center design, which may limit generalizability to diverse populations; the relatively short 6-month follow-up, potentially missing long-term biochemical dynamics; and the small sample size (n = 120), which could affect power for detecting subtle effects. Future multi-center trials with extended follow-up and additional time points are recommended.

### Conflict of interest statement

All the authors declare that they have no conflict of interest in this work.
